# PERCEIVED QUALITY OF DAILY SOCIAL INTERACTIONS: THE ROLE OF INTERACTION MODALITY

**DOI:** 10.1093/geroni/igac059.013

**Published:** 2022-12-20

**Authors:** Gizem Hueluer, Minxia Luo, Birthe Macdonald, Carlotta Grünjes

**Affiliations:** University of Bonn, Bonn, Nordrhein-Westfalen, Germany; University of Zurich, Zurich, Zurich, Switzerland; University of Zurich, Zurich, Zurich, Switzerland; University of Bonn, Bonn, Nordrhein-Westfalen, Germany

## Abstract

Older adults increasingly use digital technologies to communicate with others. In the present study, we examine the role of interaction modality (face-to-face, telephone, digital) for perceived quality of social interactions. We use data from 118 participants (age: M = 72 years, SD = 5, range = 65 to 94; 40% women), who reported on their social interactions over 21 days in an event-contingent experience sampling study. Relative to face-to-face interactions, participants reported feeling more accepted and calmer, but also less happy in telephone interactions. They perceived telephone interactions as more meaningful, but also as less pleasant. Relative to face-to-face interactions, participants felt less accepted, less close to their interaction partner, and less happy in digital interactions and they perceived digital interactions as less pleasant. In summary, our findings suggest that the modality of daily social interactions is related to their quality. We discuss implications of these findings for future research.

